# Telerehabilitation in Poststroke Anomia

**DOI:** 10.1155/2014/706909

**Published:** 2014-04-15

**Authors:** Michela Agostini, Martina Garzon, Silvia Benavides-Varela, Serena De Pellegrin, Giulia Bencini, Giulia Rossi, Sara Rosadoni, Mauro Mancuso, Andrea Turolla, Francesca Meneghello, Paolo Tonin

**Affiliations:** ^1^Foundation IRCCS San Camillo Hospital, Laboratory of Kinematics and Robotics, Neurorehabilitation Department, Via Alberoni 70, 30126 Venice, Italy; ^2^Department of Neuroscience, Azienda Ospedaliera of Padua, Italy; ^3^Neurological Rehabilitation, National Health Service, Grosseto, Italy

## Abstract

Anomia, a word-finding difficulty, is a frequent consequence of poststroke linguistic disturbance, associated with fluent and nonfluent aphasia that needs long-term specific and intensive speech rehabilitation. The present study explored the feasibility of telerehabilitation as compared to a conventional face-to-face treatment of naming, in patients with poststroke anomia. Five aphasic chronic patients participated in this study characterized by: strictly controlled crossover design; well-balanced lists of words in picture-naming tasks where progressive phonological cues were provided; same kind of the treatment in the two ways of administration. ANOVA was used to compare naming accuracy in the two types of treatment, at three time points: baseline, after treatment, and followup. The results revealed no main effect of treatment type (*P* = 0.844) indicating that face-to-face and tele-treatment yielded comparable results. Moreover, there was a significant main effect of time (*P* = 0.0004) due to a better performance immediately after treatment and in the followup when comparing them to baseline. These preliminary results show the feasibility of teletreatment applied to lexical deficits in chronic stroke patients, extending previous work on telerehabilitation and opening new vistas for future studies on teletreatment of language functions.

## 1. Introduction

Patients suffering from neurological disorders due to stroke or other brain injuries require intensive and long lasting treatments. Several national rehabilitation guidelines suggest that a reduction of hospitalization time followed by the delivery of treatment at home improves patients' outcomes and reduces costs. Nevertheless, a high percentage of discharged patients do not receive adequate motor and speech rehabilitation care, because of high staff costs and geographical barriers.

Impairment of language functions represents the second most disabling* sequela* following motor impairment and the most common cognitive deficit caused by cerebral lesions. It has been estimated that one-third of poststroke patients suffer from aphasia [1].

Aphasia is an acquired communication disorder, caused by a lesion of the brain structures involved in the processes of comprehension and production of oral and written language [2]. Lexical impairments are frequently present in aphasia and anomia is one of its principal manifestations. Anomia is associated with a breakdown in retrieving words for spoken language production. It impacts the ability to formulate sentences and participate in functional communicative exchanges, therefore, negatively conditioning the patients' quality of life as well as the lives of family members and caregivers [3].

The conventional treatment for aphasia usually begins during hospitalization in the intensive rehabilitation unit. The therapeutic management of aphasia is a long-term process that frequently does not end with a complete recovery of language and communicative functions. For many patients the progress toward functional communication is steady but slow, while other patients need to be assisted to learn compensatory strategies for an effective social communication. However, after discharge the complex* sequela* associated with stroke reduce patient's autonomy and the possibilities to reach the rehabilitation centre.

Moreover in rural areas, travel distances limit the access to specialized services affecting the quality and the quantity of rehabilitation interventions for speech disorders.

Because of the often chronic nature of the language and communicative deficits in aphasia and the difficulties that patients have in accessing specialized service providers, the ongoing management of chronic communication disorders represents a challenge for speech-language pathologists (SLPs) [7]. Recent advances in telecommunication technology have driven the development of telerehabilitation, the delivery of rehabilitation services via information and communication technologies [8] to provide rehabilitation care to patients discharged at home.

Telerehabilitation allows for continuity of service through the entire rehabilitation cycle including assessment, intervention, consultation, and education, and it has recently emerged as an effective support to provide rehabilitation care to patients discharged at home [9]. Furthermore, recent studies have shown that early discharge from hospital coupled with home treatment significantly increases clinical outcomes in poststroke patients, by affording early reintegration and positively enhancing quality of life [10, 11].

Telerehabilitation in the field of speech-language pathology has increasingly been endorsed as an appropriate model of service delivery by professional organizations in different countries. The position statement of the American speech-language and hearing association (ASHA) is that “telepractice is an appropriate model of service delivery for the professions of audiology and speech-language pathology,” because it “may be used to overcome barriers of access to services caused by distance, unavailability of specialists and/or subspecialists, and impaired mobility” and offers “the potential to extend clinical services to remote, rural, and underserved populations” [12].

Although speech-language pathology services lend themselves to telerehab applications, these are relatively new to the field of speech-language pathology. Some studies showed good reliability between conventional face-to-face and remote assessment and treatment [13, 14] on voice and articulation disturbances [15, 16]. Less studies involved aphasic deficits [17, 18] and specifically on lexical retrieval [19].

The aim of the present study was to explore the feasibility of telerehabilitation as compared with conventional face-to-face treatment of naming. Anomia treatment could be a good topic to compare the same treatment in different modalities because it allows a rigorous and controlled methodology, as well as quantitative measures of outcome.

## 2. Materials and Methods

### 2.1. Participants

We selected 5 poststroke consecutive chronic patients among 32, examined by the speech and language pathologists at IRCCS San Camillo Hospital in Venice and at Grosseto Hospital, from January to August 2013. The patients were characterized by

- having a single left ischemic stroke and aphasic disturbances persistent from 2 years or more;
- anomic deficits as evidenced by the Aachen Aphasia Test (AAT) [20] (naming less than 80% of correct items);
- comprehension good enough to understand the tasks of the study (AAT score more than 55% of correct items);
- absence of additional neuropsychological deficits (scores 30%) on attentional abilities and on nonverbal intelligence evaluated by visual search [5] and Raven's progressive matrices [6].

Patients' demographic and clinical characteristics are presented in Table 1.

The cognitive and linguistic profiles are presented in Table 2.

All subjects gave written informed consent and the study was approved by the human ethics committee.

### 2.2. Experimental Design

**Figure 1:**
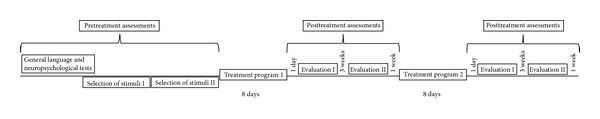
Experimental design.

The overall design of the study is shown in Figure 1. Participants first performed a baseline naming task repeated twice (see Section 2.4). Moreover, they underwent neuropsychological and linguistic assessments before entering treatment (Table 1).

Subsequently, all participants carried out two versions of the naming treatment: face-to-face treatment and teletreatment. The presentation of the first and second treatment was counterbalanced across participants in such a way that some participants began with the face-to-face treatment and some with teletreatment. There were eight sessions of equal duration for each treatment program. In each session, participants performed a confrontation-naming task whereby they were asked to name pictures of concrete objects presented on a computer screen. In the face-to-face treatment, participants sat in the room with a speech-language pathologist whereas during teletreatment the therapist communicated with the participant over the Internet. There was a three-week washout period after the first treatment cycle, during which no therapy was administered.

At the end of each treatment condition, participants' naming was assessed to determine short-term treatment efficacy. Three weeks after the completion of the treatment, participants were assessed again to determine long-term treatment efficacy (followup).

The outcome measure was naming accuracy (percent correct) on the therapy set, taken at baseline, immediately after the therapy, and three weeks later (followup). To examine for possible generalization effects to untreated items, we compared accuracy on treated items with control nontreated items. For all posttherapy measures, both treated and control items were presented in a random order. Baseline refers to naming accuracy on an individually defined set of items, which was equal to zero before treatment.

### 2.3. Neuropsychological Assessment

Standardized batteries were used to test general language and neuropsychological abilities in our patients (see Table 1). The standardized Italian version of the Aachen Aphasia Test (AAT) was used to describe patients' general language performance and the severity of anomia. Lexical ability was also assessed using the phonemic verbal fluency test (F.A.S.) and the semantic verbal fluency test [4]. To exclude additional neuropsychological deficits that could preclude the treatment, we also assessed attention by means of attentive matrices and nonverbal intelligence, by means of Raven's progressive matrices.

### 2.4. Stimuli

Two separate pretreatment baseline sessions were performed on different days. In each session, participants were asked to name a set of 255 pictures. Pictures were displayed on a computer screen for up to ten seconds, using customized presentation software (see below). If participants failed to name the picture within this time frame, the therapist advanced the trial to the next item and marked the item incorrect. Items that were scored incorrect on both pretreatment sessions were used to construct individual treatment and control lists for each participant. There were two treatment lists (face-to-face treatment and teletreatment) and two control lists (face-to-face control list and teletreatment control list) per participant. The four lists had different words and the same number of items and were balanced for word frequency, word length (in syllables), familiarity, concreteness, and age of acquisition. The words used were selected from a database for which naming norms and several psycholinguistic measures are available [21] and the pictures were colored photos.

### 2.5. Software and Materials

**Figure 2:**
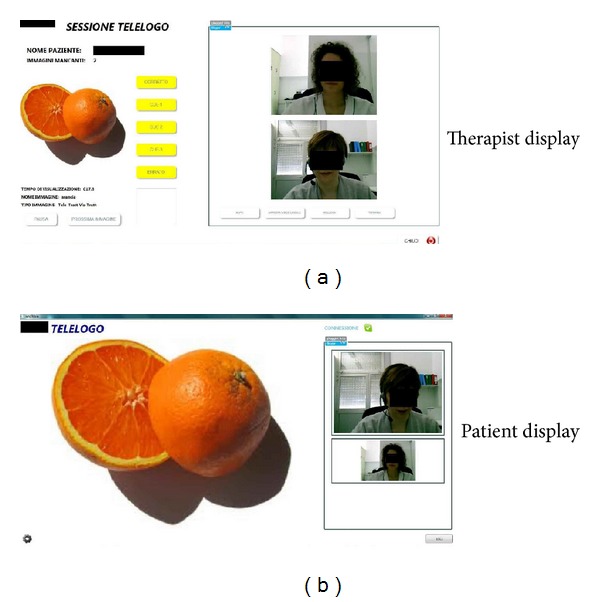
Two interfaces (one for the therapist and one for the patient) used during teletreatment.

The pictures were presented on a computer screen using customized software. The software runs on Windows 7 or XP and allows for remote communication between the therapist and the patient using an embedded Skype platform. The application displays two different interfaces: (a) the therapist's display and (b) the participant's display. The therapist's display controls and registers all experimental information including participant information, training session, and image display. The participant's display in the teletreatment condition shows 2 windows: an interactive window with the therapist (who appears on video) and a window with the target pictures (see Figure 2). In the face-to-face version, the participant's display shows only the window with the target pictures. The customized software was installed on two Intel based 17′′ laptops connected to the Internet and equipped with internal video cameras and external headphones. The Skype program was only enabled during teletreatment.

### 2.6. Treatment

During each treatment session, target pictures were presented once for naming on a computer screen in a random order. If no response was given within 10 seconds or the response was incorrect, progressive phonemic cues were provided by the therapist. A maximum of three consecutive cues were provided per picture (ranging from the initial phoneme to the full name). If the participant failed to produce the correct target word after cueing, the therapist named out loud the target asking the patient to repeat it. The same procedure was followed in the face-to-face and the teletreatment versions.

### 2.7. Statistical Analysis

Analyses of variance (ANOVAs) were used to compare naming accuracy in two treatment conditions (face-to-face and teletreatment) at three time points: baseline, after treatment, and followup (3 weeks after the end of the treatment). ANOVAs were also used to examine performance on nontreated items.

## 3. Results

All patients completed the study. Difficulties on the use of computer at home or on acceptance of teletreatment did not emerge.

### 3.1. Effects of Anomia Treatment on Treated Items

**Figure 3:**
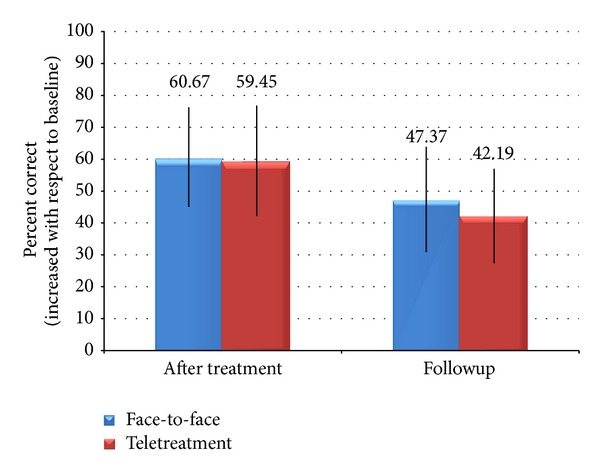
Patient's improvement on naming treated items compared to baseline naming performance.

Picture-naming performance on the treated items was evaluated before and after training. The percentage of items named correctly before and after treatment is shown in Figure 3. A 2 × 3 ANOVA with treatment type (face-to-face, teletreatment) and time (baseline, immediately after treatment, and 3 weeks after treatment) revealed a significant main effect of time (*F*(2, 24) = 11.29, *P* = .0004, and *η* ^2^ = .48), no main effect of treatment type (*F*(1, 24) = .04, *P* = .844,  *η* ^2^ = .0008), and no interaction between factors (*F*(2,24) = .02, *P* = .98, and *η* ^2^ = .0008). Post-hoc Tukey's HSD tests showed that patients performed significantly better immediately after the treatment (*P* < .01) than at baseline. There was no evidence that the treatment effect declined at followup. The patients' performance immediately after treatment did not differ from the performance 3 weeks after treatment.

### 3.2. Effects of Anomia Treatment on Control Items

**Figure 4:**
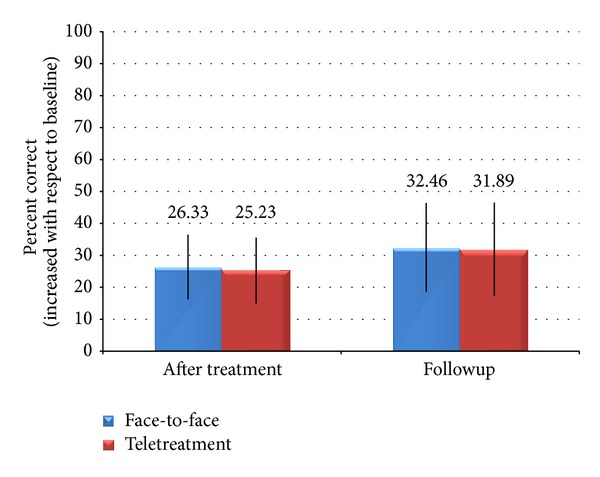
Patient's performance on nontreated items with respect to baseline performance.

Generalization of picture-naming performance on nontreated items was evaluated after training. The percentage of correct naming in posttraining measurements is shown in Figure 4. A 2 × 2 ANOVA with treatment type (face-to-face, teletreatment) and time (immediately after treatment, followup) revealed no main effect of time (*F*(1, 16) = .27, *P* = .613, and  *η* ^2^ = .016), nor treatment type (*F*(1, 16) = .004, *P* = .947, and  *η* ^2^ = .0002), and no interaction between factors (*F*(1,16) = .0004, *P* = .98, and *η* ^2^ = .00002).

In the comparison between treated and control items, we observed a main effect of item type immediately after training (*F*(1, 16) = 6.25, *P* = .02, and *η* ^2^ = .28), due to a better performance on treated (*σ* = 60.06, *δ* = 34.77) as compared to control items (*σ* = 25.78, *δ* = 21.52). There was no main effect of treatment type (*F*(1, 16) = .01, *P* = .934, and  *η* ^2^ = .0003) and no significant interaction between factors (*F*(1, 16) = .00, *P* = .996, and  *η* ^2^ = 9 × 10^−6^).

In the followup, there was no main effect of item type (*F*(1, 16) = .71, *P* = .41, and *η* ^2^ = .04), no main effect of treatment type (*F*(1, 16) = .04, *P* = .8504, and *η* ^2^ = .002), and no significant interaction between factors (*F*(1, 16) = .02, *P* = .880, and *η* ^2^ = .001).

## 4. Discussion

In this study we compared the effects of anomia therapy provided face-to-face with anomia therapy provided remotely via cost effective internet connections.

Various studies already showed the feasibility of telerehabilitation for assessment and monitoring of aphasic disorders in poststroke patients; some of these demonstrated a good reliability between face-to-face and remote consultation [7, 17, 22]. But only three previous studies were focused on a remote home based treatment of poststroke aphasic patients: two authors [19, 23] in small studies with three and two patients, respectively, highlighted a good feasibility of teletreatment, but they did not deal with the question of comparing it with a face-to-face treatment.

The third study [24], a case study on one patient, presented a comparison between the two treatment types and a third treatment based on daily independent practice.

To our knowledge, our study is the first one comparing the effects of two different treatment delivery types (remote and face-to-face), both based on the same treatment procedure and applied to a single group of patients. The selection of patients who experienced a stroke two or more years before allows us to exclude any interference of spontaneous recovery.

In the evaluations administered immediately after the treatments, we found a significant improvement on treated items in both, telerehabilitation and face-to-face conditions; we did not find differences in the percentages of correct items between the two ways of treatment.

These data confirm the feasibility of teletreatment in poststroke patients, as suggested by previous authors [13]. In particular, our results indicate that a specific treatment of lexical deficits in aphasia using tailored and controlled activities can be achieved via an Internet-based videoconferencing system.

Notwithstanding the reduced sample size of the present pilot study, we observed statistically significant effects in either type of treatment with respect to the corresponding baseline assessment and no difference between treatments. These results highlight that the teletreatment of naming deficits of poststroke chronic patients seems to be as effective as the conventional face-to-face treatment. Still, increasing the sample size and evaluating the effects of the treatments in patients with different profiles constitute avenues for future development.

In the followup evaluations, three weeks after treatments, we observed a decline of correct items, similar for both kinds of treatment, not statistically significant with respect to the posttreatment evaluations. A similar decline has been reported in previous studies [19, 25], but the short time interval between posttreatment and followup evaluations prevents us from any assumption regarding the maintenance or the loss of the improvements.

Moreover, all patients successfully completed the study and none experienced major problems in handling the system. The videoconferencing platform allowed the therapist to provide all the assistance needed, including the cues and the feedback. Thus we can consider that neither the lack of physical interaction between patient and therapist nor the technical complexities of the system hampered the effectiveness of the teletreatment.

This consideration is consistent with the conclusions of various studies on teletreatment of motor deficits after stroke, which evidenced the clinical equivalence of a remote and a face-to-face motor treatment [14, 26, 27].

Taking together these remarks, it is possible to hypothesize a future development of telerehabilitation towards more complex applications able to supply poststroke patients, early discharged at home, with a complete telerehabilitation program (motor, speech, and cognitive), similar to that received during the hospitalization.

## 5. Conclusions

Our results are encouraging and indicate that the treatment of naming deficits from remote is not inferior with respect to the conventional face-to-face treatment. These results, consistent with a few other small studies, suggest the opportunity to implement the current software also for other language components (i.e., phonology, semantics, morphology, syntax, etc.) to provide a more appropriate intervention.

## Figures and Tables

**Table 1:** Demographic and clinical characteristics of patients.

Pts	M/F	Age (yrs)	Prevalence (R/L)	Language	Lesion	Site (TAC or RMN)	Aphasia	Time from onset (yrs)
1 G.D.	M	57	R	Monolingual Italian	Ischemic	P-O	Wernicke	2
2 F.L.	M	71	R	Monolingual Italian	Ischemic	T-P	Wernicke	3
3 L.V.	M	66	R	Monolingual Italian	Ischemic	F-T-P Caudatus, Posterior Putamen	Broca	4
4 E.M.	F	63	R	Monolingual Italian	Ischemic	Insular	Broca	4
5 A.C.	M	70	R	Monolingual Italian	Ischemic	Frontal and Insular	Broca	3

**Table 2:** Linguistic and basic cognitive profile of the recruited patients (% correct).

	G.D.	F.L.	L.V.	E.M.	A.C.
AAT subtests (% correct)					
Token test	40	30	14	44	76
Repetition	44.6	78.6	72	83.3	92
Written language	63.3	94.4	7.8	18.8	80
Naming	50	52.5	13.3	54.2	79.0
Comprehension	74.2	73.3	57.5	67.5	96.6

Phonemic fluency^a^	N.A.	21	8	7	8
Semantic fluency^a^	15	13	2	10	31

Neuropsychological tests					
Attention^b^	51.7	81.7	30.0	36.7	63.3
Nonverbal intelligence^c^	77.8	86.1	36.1	47.9	39.6

^a^Phonemic and semantic fluency: raw score (Novelli et al., 1986 [4]);  ^b^Visual Search (Spinnler, 1987 [5]); ^c^Raven's progressive matrices (Carlesimo et al., 1996 [6]); NA: not applicable.
